# Biased agonism of G protein-coupled receptors as a novel strategy for osteoarthritis therapy

**DOI:** 10.1038/s41413-025-00435-y

**Published:** 2025-05-12

**Authors:** Xiangbo Meng, Ling Qin, Xinluan Wang

**Affiliations:** 1 Translational Medicine R&D Center, Institute of Biomedical and Health Engineering, Shenzhen Institutes of Advanced Technology, Chinese Academy of Sciences, Shenzhen, China; 2 University of Chinese Academy of Sciences, Beijing, PR China; 3 Musculoskeletal Research Laboratory of Department of Orthopaedics & Traumatology and Innovative Orthopaedic Biomaterial & Drug Translational Research Laboratory, Li Ka Shing Institute of Health Sciences, The Chinese University of Hong Kong, Hong Kong, China; 4 Key Laboratory of Biomedical Imaging Science and System, Chinese Academy of Sciences, Shenzhen, China

**Keywords:** Pathogenesis, Bone

## Abstract

Osteoarthritis (OA) is a prevalent degenerative joint disorder marked by chronic pain, inflammation, and cartilage loss, with current treatments limited to symptom relief. G protein-coupled receptors (GPCRs) play a pivotal role in OA progression by regulating inflammation, chondrocyte survival, and matrix homeostasis. However, their multifaceted signaling, via G proteins or β-arrestins, poses challenges for precise therapeutic targeting. Biased agonism, where ligands selectively activate specific GPCR pathways, emerges as a promising approach to optimize efficacy and reduce side effects. This review examines biased signaling in OA-associated GPCRs, including cannabinoid receptors (CB_1_, CB_2_), chemokine receptors (CCR2, CXCR4), protease-activated receptors (PAR-2), adenosine receptors (A_1_R, A_2A_R, A_2B_R, A_3_R), melanocortin receptors (MC_1_R, MC_3_R), bradykinin receptors (B_2_R), prostaglandin E_2_ receptors (EP-2, EP-4), and calcium-sensing receptors (CaSR). We analyze ligands in clinical trials and explore natural products from Traditional Chinese Medicine as potential biased agonists. These compounds, with diverse structures and bioactivities, offer novel therapeutic avenues. By harnessing biased agonism, this review underscores the potential for developing targeted, safer OA therapies that address its complex pathology, bridging molecular insights with clinical translation.

## Introduction

Osteoarthritis (OA) is a common degenerative joint disease caused by changes in the local mechanical loading, resulting in alterations in one or more signaling pathways originating from the synovial tissue, articular cartilage or subchondral bone.^1,2^ As a degenerative joint disease, it is characterized by chronic pain, restricted mobility, and reduced joint function, resulting in a significant economic impact on society and a decline in patients’ quality of life.^3^ Although OA has been commonly associated with cartilage metabolism disorders, other pathological processes such as synovial inflammation, subchondral bone remodeling imbalance, and osteophyte formation also play a role in a vicious cycle that drives disease progression.^4^ In addition, various cell types have been involved in the pathogenesis of OA, including chondrocytes, osteocytes, osteoclasts, osteoblasts, endothelial cells, immune cells, and sensory neurons.^5,6^ The disease process begins with initial cartilage damage, leading to matrix destruction and increased metabolic activity of chondrocytes. As time progresses, small cracks form in the cartilage surface, while the underlying bone plate becomes thinner and weaker. The progression of OA leads to further extracellular matrix degradation and chondrocyte senescence, resulting in deep fissures.^7^ Within the subchondral bone microenvironment, abnormal mechanical stress and pro-inflammatory mediators induce osteocytes to increase the RANKL/OPG ratio, activate osteoclasts, and stimulate bone resorption and angiogenesis.^8^ In the late stages of OA, chondrocyte death and an expansion of calcified cartilage into the superficial zone of the articular cartilage are observed. This progression is accompanied by the formation of subchondral bone cysts and the growth of sensory innervation and vascular invasion from the subchondral bone into the cartilage. Osteophyte formation is also a common occurrence in the advanced stages of OA.^9^

Clinically, patients with OA often receive palliative care, including analgesics/anti-inflammatory drugs or intra-articular corticosteroid injections for pain management.^10^ Currently, pharmacological treatments can only alleviate symptoms related to inflammation and pain, rather than cure the disease or prevent long-term disability. With an increasing understanding of OA pathology, GPCRs are being recognized as important therapeutic targets for the management of OA pain, synovial inflammation, cartilage protection, etc. Emerging disease-modifying OA drugs (DMOADs) can potentially modulate cartilage synthesis or degradation, subchondral bone remodeling, or reduce synovial inflammation to achieve therapeutic effects.^11^ Despite this, most clinical trials of DMOADs have not shown significant improvement in the pathophysiological changes of OA. Therefore, it is highly desirable to develop new treatment strategies for OA.

G protein-coupled receptors (GPCRs) are the largest superfamily of cell surface membrane receptors, encoded by approximately 1 000 genes, and sharing a conserved seven transmembrane helices (7TM).^12^ These receptors are conformationally dynamic proteins that play a critical role in mediating important biological functions in response to various extracellular signals, including photons, ions, lipids, neurotransmitters, hormones, peptides, and odorants.^13^ Due to the extensive involvement of GPCRs in the regulation of physiological processes, these receptors are of great therapeutic importance, being targeted by 30% of currently marketed pharmaceutical drugs.^14^ Many GPCRs can activate multiple intracellular signaling cascades through interactions with different types of G proteins and β-arrestins.^15^ Different agonists acting on the same GPCR can engage different effector subsets and modify cellular outcomes. This phenomenon is termed “biased signaling”.^16^ The discovery of biased ligands that favor specific signaling pathways highlights the relevance of precise control of GPCR signaling for proper therapeutic action with fewer side effects.^17,18^ In addition, the bias signaling mechanism of GPCRs is important in the pathogenesis of OA. Research indicates that certain GPCR ligands can activate specific downstream signaling pathways that affect cartilage matrix degradation, synovial membrane inflammation, subchondral bone remodeling, osteoblast formation, chondrocyte hypertrophy and maintenance of cartilage integrity, thereby influencing the progression of OA.^19^ This mechanism holds promise for the development of innovative targeted therapies for OA.

Traditional Chinese Medicine (TCM), derived from herbal and mineral sources and typically formulated as fufang, is characterized by its multiple-components and multi-target nature. TCM plays a unique role in the treatment musculoskeletal diseases.^20,21^ However, the complexity of TCM ingredients makes it difficult to evaluate efficacy using commonly used scientific methods, posing significant challenges to standardized treatment and clinical efficacy testing. Natural products derived from TCM serve as an important source for drug research and development due to their structural diversity and rich biological activities, and have shown significant potential in the treatment of OA.^22^ These natural products can be used directly as drug molecules, but also serve as lead compounds for structural optimization to develop more effective drugs with fewer side effects.^23^ For example, morphine, an alkaloid extracted from the opium herb, exerts analgesic effects by binding to μ-opioid receptors and activating G_i_ protein-mediated signaling pathways.^24^ However, it also activates β-arrestin-mediated signaling pathways, leading to adverse effects such as respiratory depression and constipation.^25^ To minimize adverse effects, researchers have developed the biased ligand TRV130 (oliceridine) for the μ-opioid receptor, which preferentially activates G protein signaling over β-arrestin-mediated signaling, thereby reducing respiratory depression and constipation compared to equianalgesic doses of morphine.^26^ Therefore, a thorough understanding of the effects of natural products associated with GPCR signaling has significant clinical implications for the development of new drugs. This article reviews the biased signaling mechanisms and biological functions of OA-related GPCRs, as well as natural products targeting these receptors, with the aim of providing a solid foundation for the development of new therapeutic drugs for OA.

## Biased agonism of G protein-coupled receptors in OA

**Fig. 1:**
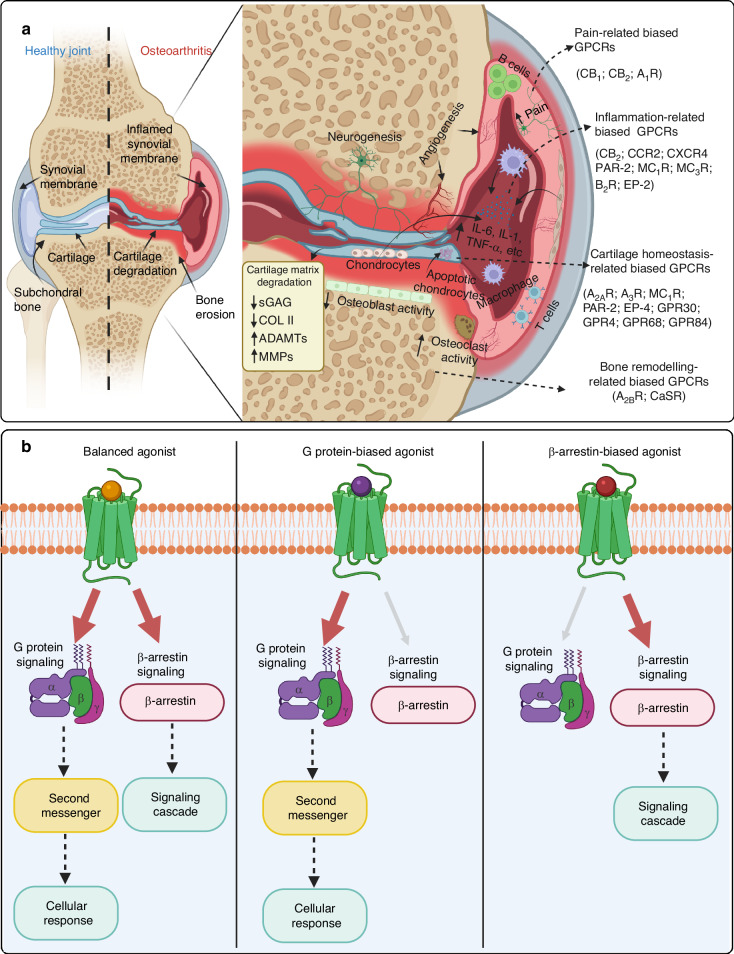
Biased G protein-coupled receptors in OA. **a** The pathogenesis of OA is closely associated with biased GPCRs, which are widely expressed across various cell types and play a crucial role in transmembrane signaling. The activated GPCRs triggers a cascade of intracellular signaling pathways that lead to a range of physiological and pathological processes, including immune cell migration, synovial inflammation, cartilage matrix degradation, cartilage angiogenesis, chondrocyte apoptosis, subchondral bone remodeling and osteophyte formation. Collectively, these processes significantly contribute to the OA progression. **b** The concept of GPCR biased signaling primarily focused on their distinct signaling mechanisms. Balanced agonists activate both G-protein and β-arrestin-dependent signaling pathways simultaneously. In contrast, G protein-biased agonists selectively engage G protein-mediated signaling pathways, influencing cellular responses through the activation of second messengers. Meanwhile, β-arrestin-biased agonists selectively activate β-arrestin-mediated signaling pathways, resulting in distinct physiological outcomes. **b** was adapted with permission from Dayoung Oh,^29^ copyright © 2021 Oliveira de Souza, Sun and Oh. (The figure was created in BioRender. M, X. (2025) https://BioRender.com/z2e00g9)

The structure and biased signaling mechanisms of GPCRs have been extensively reviewed and summarized in the literature.^15,27^ GPCRs activate various effector proteins, primarily G proteins (G_i_, G_s_, G_o_, G_q_, G_12_ and G_13_) and β-arrestins (β-arrestin 1, β-arrestin 2), which lead to the activation of downstream signaling pathways, including Ca^2+^ mobilization, cyclic adenosine monophosphate (cAMP) production, extracellular regulated protein kinase 1/2 (ERK1/2) and mitogen-activated protein kinases (MAPKs) activation. Ligands that selectively activate one signaling pathway over another are referred to as ‘biased agonism’ or ‘functional selectivity’. These ligands can be categorized into G protein-biased agonism and β-arrestin-biased agonism (Fig. 1).^28,29^ For example, the cannabinoid receptor CB_1_ can be activated by different ligands that either preferentially activate G protein signaling pathways or β-arrestin pathways. Selective activation of the G protein pathway by a ligand can result in analgesic effects without cannabis addiction associated with activation of the β-arrestin pathway. Similarly, the CB_2_ receptor can be targeted by ligands that bias towards G protein signaling to reduce inflammation and protect cartilage in OA. Recent reports have also highlighted the structure, biological functions, and the novel roles of GPCRs in the pathogenesis of OA, including cartilage matrix degradation, synovitis, subchondral bone remodeling, and osteophyte formation.^6,19^ The present article further summarizes the biased agonism of OA-related GPCRs and highlights the potential benefits of biased ligands in the treatment of OA (Table 1). In addition, clinical trials targeting GPCRs for arthritis treatment are reviewed (Table 2).

**Table 1:** Summary of the signaling mechanisms of GPCR ligands associated with OA

GPCRs	Distribution	Biased ligands	Biased signalings	Biological effects of OA treatment	Ref.
Cannabinoid receptors					
CB_1_	Central nervous Peripheral nervous	CB-05	G_i_ protein-biased allosteric modulator, inhibiting cAMP accumulation consequently decreasing neuronal excitability.	Enhancing analgesic effects and reducing cannabis addiction	^40^
CB_2_	Immune cells Osteoblasts Osteoclasts Osteocytes	JWH 133	G_i_ protein-biased agonist, mediating cAMP accumulation	Enhancing analgesic and anti-inflammatory effects and chondroprotective effects	^44^
		GW833972A	β-arrestin-biased agonist	Tolerance development	^47^
Chemokines receptors					
CCR2	Immune cells Osteoclasts Chondrocytes	J113863	Bias towards G_i/o_ signaling pathway without activating G_12_ signaling pathway	Alleviating inflammation	^60^
CXCR4	Immune cells	Monomeric CXCL12	Activates G protein-mediated cAMP and Ca^2+^ signaling pathway and recruitst β-arrestin-2	Stimulating cell migration	^72,73^
		Dimeric CXCL12	Activates G protein-mediated Ca^2+^, cAMP and ERK1/2 signaling pathway without β-arrestin recruitment	Inhibiting cell migration	
Protease activated receptors					
PAR-2	Chondrocytes Synovial cell	I-287	Bias G_q_ protein and G_12/13_ protein mediated signaling pathway and no impact G_i/o_ and β-arrestin	Alleviate inflammation	^92^
Adenosine receptors					
A_1_R	Chondrocytes Synovial cell	LUF5589	having higher efficacy for GTPγS stimulation compared to β-arrestin recruitment	Analgesic Anti-inflammatory	^98^
		VCP746	Bias G_i_ protein mediated Ca^2+^ signaling pathway		^99^
		VCP520	Bias G_i_ protein-mediated cAMP and Ca^2+^ signaling pathway		^100^
		VCP333	Bias G_i_ protein-mediated cAMP signaling pathway		
A_2A_R	Chondrocytes	Inosine	Bias towards ERK1/2 phosphorylation	Maintaining Chondrocytes homeostasis	^107^
A_2B_R	Osteoblasts Osteoclasts Osteocytes	MRS5911	Bias G_s_ protein-mediated cAMP signaling pathway	Promote osteoblast activity	^113^
		BAY60-6583	Bias G_s_ protein-mediated cAMP signaling pathway	Inhibit the osteoclast activity and reduces bone resorption	
A_3_R	Chondrocytes	MRS542 MRS1760	Activate partial β-arrestin translocation while inhibiting cAMP accumulation	Promote cell proliferation and cartilage matrix synthesis	^119^
					^120^
		LUF6000	Enhance intracellular calcium mobilization and β-arrestin recruitment		
Melanocortin receptors					
MC_1_R	Chondrocytes Synovial cells	BMS-470539	Bias towards phospho-ERK1/2	Anti-inflammation and chondroprotective effects	^130^
MC_3_R		AP1189	Bias towards phospho-ERK1/2 and calcium mobilization	Anti-inflammation	^122^
Prostaglandin E_2_ receptors					
EP-2	Chondrocytes	15-keto-PGE_2_	Binds to the EP-2 receptor, exhibiting bias towards the G_s_ protein-mediated cAMP signaling pathway	inhibits immune cell activity and reduces inflammation	^144^
Calcium-sensing receptor					
CasR	Osteoblasts Osteoclasts	NPS 2143	Negative allosteric modulators inhibit CaSR activity	Regulate calcium balance	^151^
		NPS-R568	Positive allosteric modulators promote CaSR activity		
		Cinacalcet

**Table 2:** Summarized clinical trials targeting GCPR receptors for arthritis

Target	Drug	Description	Phase	Arthritis	Treatment	Trials. gov identifier
CB_2_	GW842166	CB_2_ agonists	II	Knee OA	Administered orally	NCT00479427 NCT00447486
	LY2828360	CB_2_ agonists	II	Knee OA	Administered orally	NCT01319929
CCR2	PF-04136309	CCR2 antagonist	II	Knee OA	Administered orally	NCT00689273
A_3_R	CF101 (IB-MECA)	A_3_R agonists	II/III	Rheumatoid arthritis	Administered orally	NCT01034306 NCT02647762
B_2_R	Icatibant	B_2_R antagonist	II	Knee OA	Intra-articular injection	NCT00303056
	Fasitibant	B_2_R antagonist	II	Knee OA	Intra-articular injection	NCT02205814 NCT01091116

### Biased agonism of cannabinoid receptors in OA

Cannabinoid receptors (CBs), including CB_1_ and CB_2_, represent a class of GPCRs that specifically bind to cannabinoid compounds, including endocannabinoids, phytocannabinoids and synthetic cannabinoids, triggering signaling within cells.^30^ These receptors play a pivotal role in various physiological and pathological processes, making them promising therapeutic targets for OA.^31,32^ The recent focus on cannabinoid receptors research underscores their potential in managing OA-related pain and cartilage degradation.^33^

**Fig. 2:**
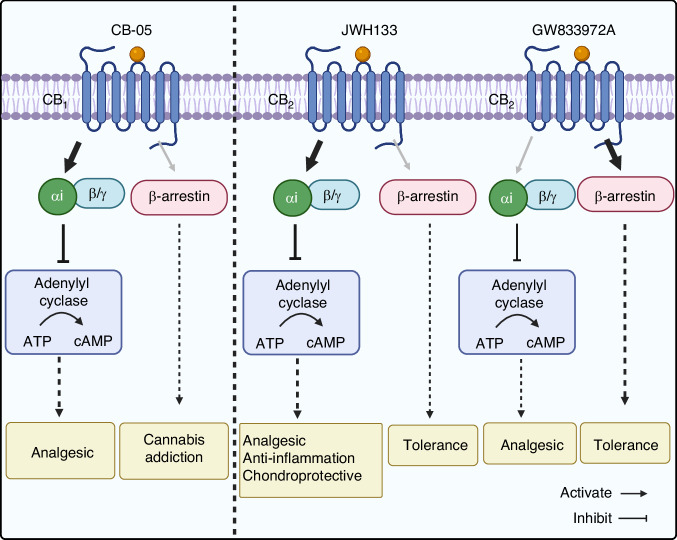
Cannabinoid receptors biased signaling mechanisms for OA treatment. The selective CB_1_ receptor agonist CB-05 primarily activates the G_αi_ protein signaling pathway rather than the β-arrestin signaling pathway, resulting in analgesic effects without inducing drug toxicity or cannabis addiction. The selective CB_2_ receptor agonist JWH133 predominantly activates the G_αi_ protein-mediated cAMP signaling pathway, demonstrating analgesic, anti-inflammatory, and chondroprotective effects. In contrast, GW833972A biases towards the β-arrestin activation pathway to achieve analgesic effects; however, its development is hindered by tolerance. (The figure was created in BioRender. M, X. (2025) https://BioRender.com/9q7zspz)

CB_1_ receptors are predominantly located in the central and peripheral nervous systems, where they modulate pain signaling.^34^ CB_1_ receptors agonists were proven to be effective analgesics in various animal models of chronic pain, including OA.^33,35^ Previous studies have demonstrated that the phytocannabinoids like Δ^9^-tetrahydrocannabinol (Δ^9^-THC) and synthetic cannabinoids (CP55940 and WIN55212-2) bind to the orthosteric site of CB_1_, activate both G-protein and β-arrestin signaling pathways, exhibiting analgesic activity.^36^ However, the activation of these pathways also poses a significant risk of cannabis addiction, limiting their clinical utility.^37^ To mitigate this risk, allosteric modulators of CB_1_ receptors have been explored.^38,39^ These modulators, such as CB-05 (the G_i_ signaling-biased agonist-allosteric modulators, ago-BAMs), selectively activate the G-protein signaling pathway while avoiding the β-arrestin pathway, demonstrating significant analgesic effects without inducing toxicity or addiction in mouse models (Fig. 2),^40^ highlighting a potential direction for future drug development.

In contrast to CB_1_, CB_2_ receptors are primarily expressed in immune cells, osteoblasts, osteoclasts, and osteocytes.^41,42^ These receptors are crucial for regulating pain, alleviating inflammation, and maintaining cartilage and bone homeostasis. CB_2_ knockout mice (CB_2_^−/−^) exhibit accelerated joint damage and diminished proteoglycan secretion in chondrocytes,^43^ suggesting a protective role for CB_2_ in OA. In contrast, overexpression of CB_2_ attenuates joint pain in monoiodoacetate (MIA)-induced OA mice,^44^ further supporting its therapeutic potential. Furthermore, CB_2_^−/−^ mice demonstrate increased osteoclast activity and accelerated bone loss.^45^ Several CB_2_ agonists have shown beneficial effects in mitigating cartilage damage, enhancing bone formation, and alleviating pain. JWH133, a selective CB_2_ agonist, biases towards G_i_-mediated cAMP signaling, protecting cartilage by downregulating matrix metalloproteinases (MMPs) and pro-inflammatory factors, while improving bone mineral density and microstructure of subchondral bone in MIA-induced OA mice.^44,46^ In contrast, GW833972A biases toward the β-arrestin activation pathway for its analgesic effects, but its development is limited due to tolerance.^47^ Similarly, HU308, another selective CB_2_ agonist, stimulates bone nodule formation in wild-type mouse osteoblasts, with no effect on CB_2_^−/−^ osteoblasts.^45^ Additional investigations using MC3T3-E1 osteoblast-like cells revealed that HU308 promotes cell migration and activates ERK phosphorylation, facilitating bone formation.^45^ Notably, while HU308 does not show a significant bias in human CB_2_ receptors, it demonstrates a pronounced bias toward G protein signaling in mouse CB_2_ receptors.^48^ This suggests that the mechanisms of action of ligands may differ considerably between species, highlighting important implications for research and drug development.

Currently, clinical trials of selective CB_2_ agonists (GW842166 and LY2828360) for OA are ongoing. GW842166 has demonstrated significant pain relief in OA patients during phase II clinical trials (NCT00479427 and NCT00447486) and has a notably lower incidence of side effects compared to non-steroidal anti-inflammatory drugs (NSAIDs), providing strong support for its clinical application. Another CB_2_ receptor agonist, LY2828360, has entered phase II clinical trials to further assess its potential for treating OA (NCT01319929). However, results indicate that LY2828360 lacks effectiveness in alleviating knee OA pain, possibly due to its complex signaling pathways.

### Biased agonism of chemokine receptors in OA

Chemokine receptors are a family of GPCRs that play a key role in leukocyte migration and inflammatory responses.^49^ They are categorized into distinct subfamilies based their preferential binding to specific chemokines: CXC chemokine receptors, CC chemokine receptors, CX3C chemokine receptors, and XC chemokine receptors.^50^ These receptors and their ligands are crucial for regulating the infiltration of inflammatory cells, chondrocyte apoptosis, and matrix degradation.^51^ In OA, the synovium releases various chemokines, such as CCL2, CCL3 and CXCL12, which bind to receptors like CCR2, CCR1 and CXCR4. This interaction leads to the infiltration of inflammatory cells, including monocytes and neutrophils, into the joint cavity.^52,53^ These cells release proteases, cytokines, and other molecules that exacerbate joint damage.^54,55^ Therefore, chemokine receptors and their associated signaling pathways are central to the pathogenesis of OA and have emerged as significant targets for OA treatment.

CCR2, widely expressed in monocytes, T cells, osteoclasts, and chondrocytes, has two primary binding sites: the orthosteric site and the allosteric site.^56,57^ The orthosteric site, located in the outer region of the transmembrane domain (TMD) of CCR2, is where the chemokine CCL2 binds.^57^ The binding induces conformational changes in the receptor, initiating G_i_ protein signaling cascades that mediate biological effects in OA, including inflammatory responses, pain and cartilage damage.^58,59^ J113863, binding to the orthosteric site of CCR2, biases toward the G_i/o_ protein signaling pathway without activating the G_12_ signaling pathway,^60^ thereby inhibiting inflammatory cell migration and exerting an anti-inflammatory effect.^61^ BMS-681, an orthosteric antagonist, primarily binds to the extracellular orthosteric pocket of the CCR2, inhibiting CCL2/CCR2 binding and reducing cytokine production associated with chronic inflammation, thereby attenuating inflammatory responses and cartilage damage.^62^ The allosteric binding site is situated at the intracellular end of the receptor TMD, can be modulated by certain small molecule compounds or peptide molecules.^57^ For instance, CCR2-RA-[R], an allosteric antagonist, binds to the intracellular allosteric pocket of the CCR2, non-competitively inhibiting CCR2 activation through direct spatial overlap with the G protein binding site.^63^ This binding obstructs the conformational changes associated with G protein binding, thereby diminishing the inflammatory response.^63^ Additionally, the CCR2 antagonist PF-04136309 has entered phase II clinical trials for OA pain, but the results remain unclear (NCT00689273).

CXCR4 is a C-X-C chemokine receptor that plays a crucial role in activation, differentiation, and migration of immune cells.^64^ It has two primary binding sites: the orthosteric binding site, which interacts with its natural ligand CXCL12/SDF-1, and the allosteric binding site, where it accommodates allosteric modulators.^65,66^ The orthosteric site is typically located between the transmembrane helix (TM) and the extracellular loop (ECL) of the receptor,^67^ while the allosteric site is positioned between the transmembrane helix (TMH) region and either the intracellular loop (ICL) or ECL.^66^ Upon binding of CXCL12/SDF-1 to CXCR4, the receptor predominantly activates the G_i_ protein, which subsequently initiates the Ras-Raf-MEK-ERK signaling pathway.^68^ The activation of ERK enhances the activity of transcription factors, such as AP-1, leading to upregulation of MMP-3, MMP-9, and MMP-13. This process contributes to cartilage matrix degradation and chondrocyte apoptosis.^69,70^ AMD3100 (Plerixafor), a CXCR4 antagonist, binds to the allosteric site of CXCR4, thereby inhibiting CXCR4-mediated signaling. This inhibition disrupts the CXCL12/CXCR4 signaling pathway, ultimately protecting chondrocytes and the cartilage matrix.^71^ In addition, several studies have found that the monomer CXCL12 secreted by cells can form dimers under physiological conditions.^72,73^ When monomeric CXCL12 binds to CXCR4, it primarily activates the G_i_ protein signaling pathway. In contrast, when dimeric CXCL12 binds to CXCR4, it predominantly promotes the recruitment of β-arrestin 2.^72^ This phenomenon illustrates a biased agonism in CXCR4 receptors, which holds significant implications for drug development targeting CXCR4.

### Biased agonism of protease-activated receptors in OA

Protease-activated receptors (PARs) are a unique family of GPCRs extensively expressed in fibroblasts, chondrocytes, osteoblasts, joint immune cells, and sensory neurons.^74^ Unlike other GPCRs, PARs are activated through proteolysis, and their signaling has been implicated in inflammation and pain associated with arthritis.^75^ PARs contain a cryptic ligand sequence at the N-terminus that is exposed upon proteolytic cleavage. This sequence can serve as a ligand and fold back into the receptor’s binding pocket, causing a conformational change that initiates an intracellular signaling cascade.^76^ Different proteases cleave PARs at various sites, leading to biased signaling.^77^ For instance, activator protein C (APC) cleaves PAR-1 at the non-canonical R^46^/N^47^ site, resulting in β-arrestin 2-mediated activation of Rac1.^78^ MMP1 cleaves PAR-1 at the non-canonical D^39^/P^40^ site, activating the G_12/13_-Rho-GTPase pathway.^79^ Trypsin cleaves PAR-2 at the canonical R^36^/S^37^ site, which triggers G_q_-mediated Ca^2+^ mobilization,^80^ along with increased Rho kinase activity via G_12/13_ signalling.^81^ Additionally, trypsin facilitates the recruitment of β-arrestin-1 and β-arrestin-2,^82^ phosphorylates ERK1/2,^83^ and promotes internalization and degradation of the receptor.^84^ In contrast, elastase cleaves PAR-2 at the S^68^/V^69^ site.^85^ Treatment of KNRK-PAR-2 cells with elastase does not induce Ca^2+^ signaling; instead, it activates ERK phosphorylation via the G_12/13_-mediated Rho kinase activation pathway.^86^ Notably, elastase does not promote β-arrestin recruitment or receptor internalization.^86^ Similarly, both neutrophil cathepsin G and proteinase 3 cleave PAR-2 downstream of the classic trypsin site (cathepsin G: P^65^/S^66^ and proteinase 3: V^62^/D^63^),^86^ but neither induces PAR-2-dependent Ca^2+^ signaling nor activates ERK phosphorylation or receptor internalization.^77^ While these proteases can deregulate PAR-2 by removing trypsin-exposed tethered ligands, it remains to be determined whether they also induce biased signaling or function as receptor antagonists. The functional relevance of PAR-2 cleavage by cathepsin G and proteinase 3 is still unclear.

PAR-2 is implicated in the pathogenesis of OA through its modulation of inflammatory responses in cartilage and synovium, as well as its influence on the balance between cartilage matrix breakdown and synthesis.^87,88^ Husa et al. investigated OA models of wild-type (WT) and PAR-2 deficient (PAR^2-/-^) mice, revealing that PAR-2 promotes cartilage proliferation and hypertrophy, ultimately contributing to osteophytes formation.^89^ The study suggests that abnormal activation of PAR-2 may be linked to arthropathy and could potentially enhance the onset and progression of OA. PAR-2 is activated by various proteases (trypsin, thrombin, and elastase) and couples with G_q/11_ G_i/o_ and G_12/13_ protein, triggering multiple intracellular signaling cascades.^90^ These cascades activate several signaling pathways, including phospholipase C-β (PLCβ), inositol 1,4,5-trisphosphate/diacylglycerol (IP_3_/DAG), Protein kinase C (PKC), Nuclear factor-κB (NF-κB), and Mitogen-activated protein kinase (MAPK). Upon activation, PAR-2 promotes the expression and release of inflammatory mediators such as IL-1β, TNF-α, IL-6, and PGE_2_,^91^ which exacerbate synovial inflammation. Furthermore, activated PAR-2 facilitates the upregulation of MMPs, including MMP-1, MMP-3, and MMP-13,^88^ leading to the degradation of cartilage matrix components. Therefore, inhibiting PAR-2 activity may be an effective strategy to alleviate the progression of OA. Charlotte et al. identified a novel selective and potent PAR-2 inhibitor, I-287, which acts as a negative allosteric modulator on G_q_ and G_12/13_ activities while showing no impact on G_i/o_ signaling and β-arrestin 2 engagement.^92^ This specific inhibition of select PAR-2 pathways effectively blocked inflammation in vivo. In a separate study, Huang et al. discovered that the PAR-2 antagonist AZ3451 mitigated chondrocyte apoptosis by activating autophagy and exerted chondroprotective effects through the regulation of the P38/MAPK, NF-κB and PI3K/AKT signaling pathways.^93^

In summary, PAR-2 plays a significant role in the pathogenesis of OA through multiple mechanisms, including the regulation of inflammatory responses, the promotion of a balance between cartilage matrix degradation and synthesis, and the modulation of chondrocyte proliferation and apoptosis. The development of selective inhibitors targeting PAR-2, such as I-287 and AZ3451, show promise for the treatment of OA and offer new directions for future therapeutic strategies.

### Biased agonism of adenosine receptors in OA

Adenosine receptors (ARs) are a class of GPCRs that primarily include A_1_R, A_2A_R, A_2B_R, and A_3_R. These receptors play a crucial role in regulating various physiological functions, including nerve conduction, immune response, and cell metabolism, through their interaction with the endogenous molecule adenosine. In OA, the activation of ARs is significant for multiple physiological processes, including pain management, chondrocyte senescence, apoptosis, autophagy, and inflammatory responses.

A_1_R is expressed in both the peripheral and central nervous systems.^94^ The activation of A_1_R has been shown to alleviate pain in monosodium iodoacetate induced OA rats.^95^ When activated, A_1_R inhibits adenylyl cyclase through inhibitory G_i/o_ proteins, resulting in the activation of inwardly rectifying K^+^ channels.^96^ This process suppresses neurotransmitter release across the synapse, thereby alleviating pain transmission.^97^ The A_1_R contains both orthosteric and allosteric sites, which can be targeted by biased agonists. For example, LUF5589, a potential biased agonist, binds to the orthosteric site and demonstrates higher efficacy for [35S] GTPγS stimulation compared to β-arrestin recruitment in U2OS cells.^98^ VCP746, another biased agonist of A_1_R, can simultaneously engage the orthosteric site and allosteric site.^99^ This compound demonstrates significant functional selectivity toward calcium mobilization, which contribute to its cytoprotective properties.^99^ Positive allosteric modulators (PAMs) of A_1_R, such as VCP520 and VCP333, influence receptor activity by targeting allosteric sites, which are non-primary binding sites on the receptor.^100^ These PAMs exhibit distinct bias patterns; for instance, VCP333 preferentially increases cAMP levels, inhibiting neuronal excitability and reducing the transmission of pain signals, while VCP520 modulates calcium channel activity, thereby reducing excessive neuronal excitation and aiding in pain relief.^100^

**Fig. 3:**
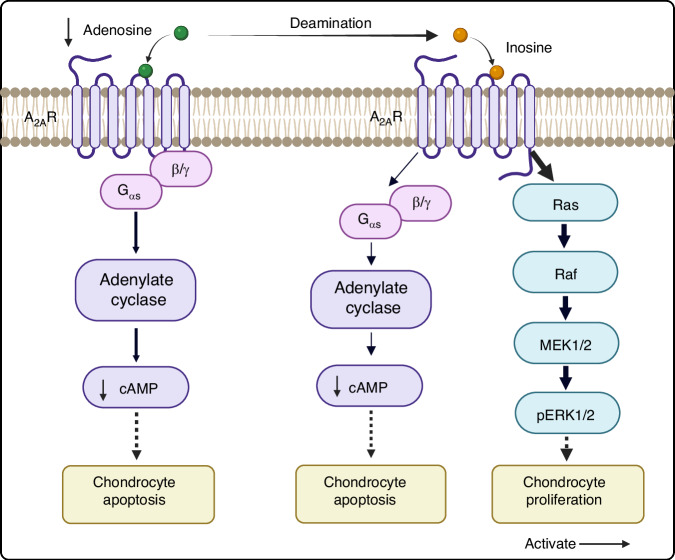
This schematic illustrates A_2A_R in OA chondrocytes. In OA chondrocytes, there is a decrease in ATP production, reduced utilization of exogenous adenosine, and a decline in cAMP levels, all contributing to increased chondrocyte apoptosis. In contrast, inosine, a stable decomposition product of adenosine, biases toward ERK1/2 phosphorylation, enhances chondrocyte proliferation, and inhibits chondrocyte apoptosis. (The figure was created in BioRender. M, X. (2025) https://BioRender.com/es1159u)

The A_2A_R plays a crucial role in maintaining chondrocyte homeostasis.^101^ Previous studies have reported that the mice lacking A_2A_R spontaneously develop OA at 16 weeks of age due to the loss of adenosine signaling, which results from decreased adenosine production.^102,103^ This observation parallels findings in human OA.^103^ In OA chondrocytes, ATP production is diminished, and the availability of exogenous adenosine is also reduced (Fig. 3).^104^ This leads to decreased stimulation of A_2A_R, disrupting the chondrocytes homeostasis and thereby promoting the OA progression.^104^ These findings suggest that activation of A_2A_R can mitigate chondrocyte senescence and promote the formation of the anti-aging p53 variant Δ133p53α.^105,106^ Additionally, A_2A_R stimulation enhances autophagic flux, increases the activation and nuclear localization of FoxO1 and FoxO3, improves the metabolic function in chondrocytes, and reduces markers of apoptosis.^102^ The activation of A_2A_R can enhance mitochondrial metabolism and reduce mitochondrial damage mediated by reactive oxygen species, a phenomenon verified in an obesity-induced OA mouse model.^104^ Intra-articular injection of liposomal A_2A_R agonists, such as CGS21680, significantly promotes cartilage formation and enhances cartilage homeostasis, thereby reducing chondrocyte senescence.^105^ Furthermore, inosine, a stable breakdown product of adenosine, activates A_2A_R with a bias towards ERK1/2 phosphorylation rather than cAMP accumulation (Fig. 3).^107,108^ This mechanism may promote chondrocyte proliferation and inhibit chondrocyte apoptosis.^108,109^ Therefore, inosine exhibits superior stability and bioavailability compared to adenosine, and its distinct signaling properties confer unique advantages in both physiological and pathological contexts. These attributes may establish inosines as a pivotal molecule for clinical treatment and drug development.

A_2B_R is closely associated with the maintenance of bone homeostasis. When A_2B_R are activated, it promotes the differentiation of mesenchymal stem cells into osteoblasts, enhances the ability for bone formation, and facilitates the synthesis and mineralization of the bone matrix, thereby contributing to the formation of new bone.^110^ Carroll et al. found that A_2B_R knockout mice exhibited enhanced osteoclast activity, reduced bone density, and increased bone resorption.^111^ This phenomenon indicates that the A_2B_R plays a crucial role in bone metabolism by inhibiting osteoclast activity and promoting osteoblast function. However, A_2B_R exhibits a lower affinity for adenosine compared to other adenosine subtypes.^112^ Consequently, researchers have been actively seeking highly selective and effective biasing and allosteric agonists. MRS5911 and BAY60-6583 are identified as biased agonists of A_2B_R, preferentially promoting cAMP accumulation over ERK1/2 phosphorylation or calcium mobilization.^113^ Additionally, BAY60-6583 inhibits RANKL-mediated upregulation of osteoclast marker genes, leading to reduced fusion of osteoclasts and decreased bone resorption activity.^114^ Notably, capadenoson, an A_1_R partial agonist, shares a structural similarity with BAY60-6583,^115^ also acts as a biased A_2B_R agonist that significantly favors cAMP accumulation. These promising findings underscore the necessity for further research to elucidate the therapeutic potential of biased and allosteric A_2B_R agonists in the treatment of bone diseases.

The activity of the A_3_R in chondrocytes is intricately associated with the OA pathogenesis. Genetic ablation of the A_3_R leads to articular cartilage degeneration in aged mice. Mechanistically, A_3_R signaling inhibits cellular catabolic processes in chondrocytes by downregulating calcium/calmodulin-dependent protein kinase II (CaMKII), an enzyme known to promote matrix degradation and inflammation, as well as Runt-related transcription factor 2 (RUNX2).^116^ A_3_R activation have demonstrated the ability to protect chondrocytes from apoptosis induced by inflammatory factors while concurrently reducing cartilage matrix degradation.^117^ These findings underscore the potential protective role of A_3_R in maintaining articular cartilage homeostasis and in preventing the progression of OA. CF101(IB-MECA), an A_3_R agonist, could protect articular cartilage against OA by enhancing the ratio of ATP/AMP and altering the AMPK/mTOR pathway to enhance autophagy and reduce inflammation.^118^ In addition, CF101 shows anti-inflammatory and analgesic potential in preclinical and phase II/III clinical trials (NCT01034306 and NCT02647762). Moreover, MRS542 and MRS1760 are biased A_3_R ligands and activate partial β-arrestin translocation while inhibiting cAMP accumulation.^119^ Furthermore, allosteric modulators like LUF6000 can selectively enhance intracellular calcium mobilization and β-arrestin recruitment without influencing the responses of MRS542 in cAMP and membrane hyperpolarization.^120^

### Biased agonism of melanocortin receptors in OA

Melanocortin receptors (MCR) are seven-transmembrane (TM) domain proteins that are coupled to G-proteins and signal through intracellular cyclic adenosine monophosphate (cAMP).^121^ There are five subtypes of MCR: MC_1_R, MC_2_R, MC_3_R, MC_4_R, and MC_5_R. Each subtype exhibits a distinct tissue expression pattern and varies in the relative potency of different melanocortin peptides. Various orthogonal binding site and allosteric regulatory sites are present in MCR.^121^ The orthogonal binding site is situated in the transmembrane region of the receptor and binds natural peptide ligands like α-melanocyte stimulating hormone (α-MSH). Apart from the orthogonal binding site, MCR may also feature allosteric sites, which play an important role when the receptor structure changes and can affect the activity and signaling efficiency of the receptor.^122^ Different biased ligands selectively bind to various sites on MCRs, thereby influencing receptor signal transduction pathways. NDP-MSH ([Nle⁴, D-Phe⁷]α-MSH), for instance, serves as a selective agonist that can engage with the orthogonal binding sites of MC_1_R, MC_3_R, MC_4_R, and MC_5_R, activating the cAMP pathway.^123^ In contrast, HS014 is a highly specific antagonist of MC_4_R, which inhibits cAMP signaling.^124^ Furthermore, allosteric modulators such as ML00253764 modulate receptor conformation by binding to the allosteric site of MC_4_R, thereby impacting the efficiency and bias of signal transduction.^125^ BMS-470539 is a highly selective agonist of MC_1_R that does not activate any other melanocortin receptors.^126^ It regulates transcription factors via the cAMP-PKA pathway and contributes to the stabilization and repair of the actin cytoskeleton.

Recent studies suggest that MCRs are associated with the progression of OA.^127^ Melanocortin peptide pretreatment has been shown to prevent chondrocytes apoptosis, reducing in pro-inflammatory cytokines and promoting the production of the anti-inflammatory cytokine.^128^ MC_1_R deficient mice exhibit articular cartilage damage and elevated inflammatory cytokines, resulting in an OA-like phenotype.^129^ Specifically, the absence of the MC_1_R accelerates age-related changes in the cartilage matrix, characterized by a decrease in type II collagen (Col II) and an increase in MMP-13-positive chondrocytes.^127^ Activating MC_1_R can induce aging in synovial tissue and provide protection to cartilage in vivo, thereby exerting anti-arthritic effects.^129^ Given the critical role of MC_1_R in OA, the agonist BMS-470539 was found to bias ERK1/2 phosphorylation and demonstrated significant anti-inflammatory and chondroprotective effects on lipopolysaccharide (LPS) -induced chondrocytes.^130^ AP1189, an orally bioavailable small molecule ligand, biases towards ERK1/2 activation and calcium mobilization of MC_3_R in HEK293A cells, but not activate Gas-cAMP levels.^122^ Research indicates that AP1189 exerts anti-inflammatory effects through MC_3_R-mediated ERK1/2 phosphorylation, underscoring its potential for regulating inflammatory responses and for the development of novel anti-inflammatory drugs.

### Biased agonism of bradykinin receptor in OA

Bradykinin is a small endogenous proinflammatory peptide known to be an effective inducer of acute pain. It is a peptide consisting of nine amino acids that belongs to the kinin family and plays a crucial role in cardiovascular homeostasis, pain and inflammation.^131^ There are two bradykinin receptor subtypes, B_1_ receptor (B_1_R) and B_2_ receptor (B_2_R).^132^ B_2_R is continuously expressed in normal tissues and mediates many of the acute effects of kinins, whereas B_1_R is more closely associated with chronic responses in inflammation.^133^ Both receptors can interact with the G_i_ and G_q_ families of G proteins, initiating secondary signaling cascades that involve molecules such as PLC, PKC, Ras/Raf-1/MAPK, and PI3K/AKT, as well as secondary messengers like IP_3_, DAG, and Ca^2+^.^131^ These secondary messengers regulate the production of inflammatory mediators, including NO, arachidonic acid, prostaglandins, and leukotrienes, ultimately leading to the release of additional inflammatory factors.^133^

B_2_R have been found in synovial cells, fibroblasts, and chondrocytes in OA patients,^134^ while B_1_R has been found in rheumatoid arthritis synovial tissue and fibroblast-like synoviocytes (FLSs).^135^ Activated B_2_R can initiate signaling cascades that lead to painful and inflammatory responses and thus might potentially contribute to cartilage degradation in OA pathology.^134^ These findings suggest that B_2_R antagonists may be beneficial in treating OA. Icatibant is a synthetic decapeptide and antagonist of B_2_R.^136^ Clinical study have shown that icatibant significantly reduces pain intensity in patients with OA (NCT00303056).^137^ Fasitibant, another small molecule B_2_R antagonist, has demonstrated the ability to inhibit the inflammatory response of human synovial fibroblasts, particularly the release of interleukin-6 (IL-6) and interleukin-8 (IL-8) induced by bradykinin.^138^ Fasitibant has entered clinical trials to evaluate its effectiveness in alleviating OA symptoms (NCT02205814 and NCT01091116).

### Biased agonism of prostaglandin E_2_ receptors in OA

Prostaglandin E_2_ (PGE_2_) is a significant organic compound synthesized from arachidonic acid (AA) in vivo through the action of cyclooxygenase (COX).^139^ In OA patients, the synovium and cartilage produce elevated levels of PGE_2_, which serves as a key pro-inflammatory pain mediator.^140^ There are four primary subtypes of PGE_2_ receptors: EP-1, EP-2, EP-3, and EP-4.^141^ Prostaglandin receptors can be activated not only by their specific ligands but also by noncognate prostaglandins, which act as biased ligands.^142,143^ For instance, PGE_1_ and PGE_3_ function as negatively biased agonists of the EP-4, exerting anticancer effects by partially activating EP4-mediated β-catenin/TCF signaling. These biased activities may arise from distinct receptor conformations caused by the number and pattern of hydrogen bond formations between the EP-4 and each ligand.^143^ 15-keto-PGE_2_ is a metabolite of PGE_2_ that is typically generated later in the inflammatory response. It binds to EP-2 to attenuate and/or terminate PGE_2_-induced pain or inflammation.^144^ Specifically, 15-keto-PGE_2_ binds to the EP-2, exhibiting bias towards the G_s_ protein-mediated cAMP signaling pathway, which inhibits immune cell activity and reduces inflammation. In addition, several studies have indicated that elevated levels of PGE_2_ are closely associated with cartilage degeneration and subchondral bone remodeling in OA. PGE_2_ can inhibit the synthesis of proteoglycans in cartilage *via* the EP-4 receptor and promote the expression of MMPs and aggrecanase-degrading articular cartilage matrix-5 (ADAMTS-5).^145^ Furthermore, PGE_2_ enhances angiogenesis in subchondral bone and stimulates the innervation of sensory neurons by activating EP-4 receptors on osteoclasts, thereby aggravating OA progression and pain.^146^ The EP-4 antagonist HL-43 specifically blocks EP-4 receptors and inhibits PGE_2_-induced osteoclast activation and cartilage degradation, thereby slowing the progression of OA.^147^ Additionally, HL-43 reduces H-type blood vessel formation and Netrin-1 secretion in subchondral bone, alleviates pain sensitivity, and regulates the function and activity of osteoclasts through the modulation of the G_s_/PI3K/AKT/MAPK signaling pathway.^146^

In summary, PGE_2_ and its receptors are intricately involved in the pathophysiology of OA, influencing cartilage metabolism, subchondral bone remodeling, and pain perception. Intervention strategies that target PGE_2_ and its receptors, particularly the EP-4 receptors, may open new avenues for OA treatment and enhance patients’ joint function and quality of life. Future research should investigate the mechanisms underlying PGE_2_ receptor action in OA and explore effective methods to regulate these receptors to slow disease progression.

### Biased agonism of calcium-sensing receptors in OA

Calcium-sensing receptors (CaSR) are GPCRs that play an important role in maintaining calcium and magnesium homeostasis as well as in the secretion of parathyroid hormone.^148,149^ The CaSR contains multiple calcium ion binding sites for endogenous and exogenous ligands, along with several allosteric sites.^150^ When CaSR is activated, it initiates different signaling pathways primarily through three types of heterotrimeric proteins: G_q/11_, G_i/o_ or G_12/13_.^151^ This activation leads to biased agonism, where different ligands preferentially stimulate specific downstream signaling responses of CaSR.^152^ Additionally, CaSR-ligand interactions can be modulated by positive allosteric modulators (PAMs) and and negative allosteric modulators (NAMs). PAMs, such as NPS-R568 and cinacalcet, enhance CaSR activity, leading to increased blood calcium levels and treat conditions like osteoporosis.^153^ In contrast, NAMs such as NPS-2143 inhibit CaSR activity, reducing sensitivity and aiding in the treatment of hypercalcemia-related disorders.^154^ In addition, magnesium can bind to CaSR, triggering intracellular calcium signaling and the phosphorylation of ERK1/2, which promotes osteoblast differentiation and new bone formation.^155,156^

Studies have shown that the CaSR has various effects on chondrocytes and osteocytes.^151^ The absence of CaSR is associated with abnormal cartilage calcification, growth plate abnormalities, and defects in bone development.^157^ Moreover, research indicates that CaSR deletion can negatively impact bone mass and osteocyte survival,^158^ suggesting that targeting CaSR in osteoblasts/osteocytes could result in bone anabolic effects. For instance, strontium-induced osteoclast apoptosis activates the anabolic Wnt pathway and promotes Akt phosphorylation (p-AKT) downstream of CaSR in osteoblasts.^159^ Additionally, it encourages osteoblastgenesis while inhibiting osteoclastogenesis by blocking NF-κB activation.^160^ Cinacalcet, an FDA-approved small molecule PAM of CaSR, is used for the treatment of primary and secondary hyperparathyroidism. It reduces the activity of tartrate-resistant acid phosphatase (TRAP), thereby impairing human osteoclast’s function and significantly affecting bone absorption.^161^ Furthermore, biomechanical stress has been found to upregulate CaSR expression, which plays a crucial role in chondrocyte terminal differentiation. Local administration of the CaSR antagonist NPS2143 has shown promise in halting the progression of OA.^162^ Additionally, Mg^2+^ and tryptophan derivative (L-1,2,3,4-tetrahydronorharman-3-carboxylic acid, TNCA), can cooperatively activate CaSR, inhibit chondrocyte apoptosis, promote the synthesis of cartilage matrix, and prevent the OA progression.^155,163^ Mg^2+^ supplementation may thus offer a novel therapeutic approach for OA patients and demonstrates promising clinical application prospects.

### Other GPCRs in OA

In cartilage, GPCRs influence the chondrocytes senescence and the synthesis of the cartilage matrix by mediating signals from hormones, neurotransmitters, inflammatory mediators and so on. Bai et al. utilized a high-throughput drug screening system to demonstrate that the α-adrenoceptor inhibitor phentolamine can simultaneously induce chondrogenesis and inhibit cartilage hypertrophy. In vivo experimental results indicate that phentolamine promotes the differentiation of endogenous stem cells into hyaline cartilage while inhibiting the formation of fibrocartilage in a mouse cartilage defect model.^164^ Additionally, the activation of some GPCRs can influence the formation of extracellular matrix. GPR30 activation has been shown to inhibit ferroptosis and protect chondrocytes from OA. Notably, the expression of GPR30 in OA cartilage tissue is lower than that in normal tissue, and its activation inhibits ferroptosis in chondrocytes by suppressing YAP1 phosphorylation, which regulates FTH1 expression.^165^ Furthermore, another study revealed that the proton-activated G protein-coupled receptor GPR4 is critical for the development of OA. GPR4 activates the NF-κB/MAPK signaling pathway by regulating the expression of CXCL12, inhibits chondrocyte differentiation, and upregulates cartilage homeostasis.^166^ Inhibition of GPR4 with the antagonist NE52-QQ57 can ameliorate the progression of OA in mice, promote extracellular matrix production, and protect cartilage degradation.^166^ GPR68, a pH-sensing GPCR, can be activated in acidic environments and regulates intracellular signaling through interactions with G proteins.^167^ The activation of GPR68 can inhibit the MMPs expression in OA chondrocytes, suggesting that it may serve as a potential target for the treatment of OA.^168^ GPR84, a receptor for medium-chain fatty acids (MCFAs), is the only fatty acid-sensing GPCR in human and mouse chondrocytes that exhibits elevated expression when stimulated by IL-1β. The deficiency of GPR84 resulted in an increased expression of cartilage catabolic regulators and a decreased expression of anabolic factors in the IL-1β-induced cell model and the destabilization of the medial meniscus - induced OA mouse mode.^169^ Furthermore, the activation of GPR84 can enhance the production of cartilage extracellular matrix, whereas the agonists of GPR84 protected human OA cartilage against degeneration by inducing cartilage anabolic factor expression.^169^

## Natural products derived from TCM targeting G protein-coupled receptor in OA treatment

TCM has accumulated valuable clinical experience over thousands of years of application, especially for bone and joint diseases.^22^ However, the characteristic of multiple components acting on various targets present challenges in evaluating the efficacy of TCM.^170^ With advancements in science and technology, researchers have extracted numerous natural products from TCM, which are considered significant natural sources with a wide range of therapeutic potential. For instance, morphine, an alkaloid extracted from the opium herb, is widely used as an anesthetic and analgesic drug, thereby facilitating human exploration of natural products.^171^ In drug development, alkaloids, terpenes, and flavonoids are the most common chemical structures that target GPCRs for the OA treatment (Table 3).^172^

**Table 3:** Natural products derived from TCM targeting G protein-coupled receptor in OA treatment

Natural products	Source	Chemical structures	Binding receptors	Biological effects of OA treatment	Ref.
Alkaloids					
Sinomenine	*Sinomenium acutum* (Thunb.) Rehd. et Wils.		A_2A_R MRGPRX2	Inhibit NF-κB activation to alleviate arthritis	^175^
Berberine	*Coptis chinensis* Franch.		CCR2	Blocks the MCP-1/CCR2 signaling pathway by binding to CCR2, which reduces the migration of inflammatory cells	^177^
Terpenoids					
Paeoniflorin	*Paeonia lactiflora* Pall.		A_1_R	Inhibited expression of MMPs and pro-inflammatory mediators	^180,181^
Andrographolide	*Andrographis paniculata* (Burm. f.) Wall. ex Nees in Wallich		A_2A_R	Anti-inflammatory and Antioxidative	^182,183^
Celastrol	*Tripterygium wilfordii* Hook. f.		CB_2_	Inhibit cAMP accumulation	^184,185^
Acetyl-11-keto-β-boswellic acid	*Boswellia serrata* Roxb. ex Colebr.		CXCR4	Inhibited inflammation and extracellular matrix degradation. Alleviated OA progression via the Nrf2/HO-1 pathway	^186,187^
Astragaloside IV	*Astragalus membranaceus* (Fisch.) Bunge		CXCR4	Inhibit ADAMTS-4, ADAMTS-5 overexpression in chondrocytes through inhibiting PI3K-Akt signaling pathway	^188^
Flavonoids					
Biochanin A	*Trifolium pratense* L.		GPR30	Inhibit the release of inflammatory cytokines	^193^
Baicalin	*Scutellaria baicalensis* Georgi.		A_2A_R	Inhibit LPS-induced inflammatory response and protect the chondrocytes’ function	^194^

### Alkaloids targeting GPCRs

Alkaloids, a class of nitrogen-containing basic organic compounds, are commonly utilized in TCM for the prevention and treatment of OA.^173^ These compounds regulate cell morphology, apoptosis, and autophagy. Sinomenine (SIN), a natural product derived from *Sinomenium acutum* (Thunb.) Rehd. et Wils., has been shown to possess potent anti-inflammatory and cartilage protective properties. In mouse cartilage cells, SIN has been observed to inhibit the inflammatory response and ECM degradation by activating the Nrf2/HO-1 signaling pathways while inhibiting NF-κB activity in mouse cartilage cells.^174^ The molecular mechanism of action of SIN involves binding to various GPCRs, with the most notable being the A_2A_R and the MRGPRX2. SIN has been shown to inhibit the NF-κB pathway upon binding adenosine A_2A_R, thereby reducing symptoms associated with arthritis.^175^ However, SIN has also been observed to interact with MRGPRX2, leading to Ca^2+^ mobilization in mast cells via the PLC-IP_3_-Ca^2+^ pathway, which subsequently triggers mast cell degranulation and adverse reactions.^176^ Berberine, an alkaloid from *Coptis chinensis* Franch, blocks the MCP-1/CCR2 signaling pathway by binding to CCR2, thereby reducing the migration of inflammatory cells such as monocytes and macrophages.^177^ Additionally, berberine increases the level of proteoglycans in cartilage matrix and enhances the thickness of articular cartilage, as evidenced by elevated expressions of Col II, p-Akt and phosphorylated S6 protein (p-S6) in a rat OA model.^178^

### Terpenoids targeting GPCRs

Terpenoids represent fundamental compounds involved in plant growth and metabolism.^179^ Numerous studies have highlighted the significant anti-osteoarthritic effects of terpenoids, including monotepenes, diterpenes, triterpenes and so on. Paeoniflorin, a monoterpene from *Paeonia lactiflora* Pall., has been shown to bind to A_1_R, promoting the phosphorylation of AKT and ERK1/2,^180^ and reducing the expression of inflammatory mediators, including IL-1β, IL-6, and TNF-α, in LPS-induced OA. Furthermore, the inhibition of MMP13 and ADAMTS-5 expression contributes to the reduction of the inflammatory response, thereby offering protection to cartilage.^181^ Andrographolide (AP), a diterpene derived from *Andrographis paniculata* (Burm. f.) Wall. ex Nees in Wallich, binds to the adenosine A_2A_R, leading to an increased formation of cAMP through the G_s_ protein-mediated signaling pathway.^182^ This elevation in cAMP activates protein kinase A (PKA), which subsequently inhibits the activity of glycogen synthase kinase-3β (GSK-3β) through PKA mediated phosphorylation. As a result, this leads to the sustained activation of nuclear factor E2-related factor 2 (Nrf2) within the nucleus and enhances the expression of heme oxygenase-1 (HO-1).^183^ In addition, AP protects chondrocytes from oxidative stress damage via activation of the Keap1-Nrf2-Are pathway in H_2_O_2_-induced chondrocytes.^183^ Celastrol, extracted from *Tripterygium wilfordii* Hook. f., blocks the NF-κB signaling pathway, promotes the activation of autophagy, and attenuates the apoptosis of chondrocytes.^184^ Jiang et al. found that celastrol can bind to the CB_2_, regulating the phosphorylation of ERK1/2 via β-arrestin2 mediated signaling pathway, which in turn inhibits the inflammatory response.^185^ Acetyl-11-keto-β-boswellic acid (AKBA), the active compound of *Boswellia serrata* Roxb. ex Colebr., binds to CXCR4 and activates the MAPK and PI3K/AKT signaling pathways via G_i/o_ protein-mediated signaling pathway.^186^ This action regulates the mobilization of Ca^2+^ in leukocytes, enabling AKBA to modulate immune and inflammatory responses.^187^ Astragaloside IV (ASN IV), the primary phytochemical in *Astragalus membranaceus* (Fisch.) Bunge, has been identified as a novel CXCR4 antagonist. Yang et al. show that ASN IV decreases the overexpression of ADAMTS-4 and ADAMTS-5 in chondrocytes by inhibiting the CXCL12/CXCR4 signaling pathway.^188^ Furthermore, administration of ASN IV effectively repaired cartilage and subchondral bone damage in MIA-induced OA rats.^188^ ASN IV also inhibits IL-1β-induced inflammatory response in human OA chondrocytes and ameliorates the progression of OA in mice.^189^

### Flavonoids targeting GPCRs

Flavonoids, a class of natural polyphenolic compounds found in plants, have garnered significant attention for their anti-inflammatory and antioxidant properties.^190^ Studies indicate that flavonoids show promise in treating OA by mitigating the inflammatory processes linked to arthritic lesions.^191^ These compounds function by inhibiting the release of inflammatory mediators in joint tissues, thus potentially aiding in the alleviation of OA.^192^ Biochanin A (BCA), derived from *Trifolium pratense* L., has the ability to bind to GPR30 and trigger GPR30-mediated signaling, resulting in neutrophil apoptosis and inhibition of inflammation via the cAMP/PKA signaling pathway.^193^ Baicalin, derived from *Scutellaria baicalensis* Georgi, binds to A_2A_R at specific locations, can inhibit LPS-induced inflammatory response and protect the chondrocytes’ function.^194^

### Pros and cons of natural products targeting GPCRs in OA treatment

Natural products derived from TCM serve as a vital resource for innovative drug discovery^195^ in OA treatment, offering structurally diverse lead compounds and candidate drugs with broad biological activities, particularly in modulating GPCR signaling pathways-a key therapeutic target for OA-related inflammation and cartilage degeneration.^172^ Advantages include their multi-target potential, which may synergistically regulate interconnected pathways (e.g., suppressing inflammatory cytokines via CB_2_ receptor activation while enhancing chondrocyte survival). However, limitations arise from poor drug-likeness, such as low bioavailability, narrow therapeutic indices, and weak target specificity, which often lead to off-target effects or diluted therapeutic outcomes. Recent advances in AI-driven molecular design,^196^ high-throughput screening,^197^ and structural biology have enabled the rational optimization of these natural products. For instance, structural refinement of TCM-derived compounds has yielded biased GPCR modulators (e.g., β-arrestin-biased ligands), which selectively activate anti-inflammatory pathways while minimizing G protein-mediated side effects (e.g., gastrointestinal toxicity). These innovations address the inherent challenges of natural products, enhancing their efficacy and safety profiles in OA therapy and bridging traditional medicine with modern precision pharmacology.

## Future perspectives and conclusions

G protein-coupled receptors (GPCRs) have emerged as pivotal therapeutic targets in OA, yet their diverse signaling mechanisms demand a nuanced approach. Cannabinoid receptors exemplify the dichotomy of therapeutic potential and limitations: while CB_1_ agonists offer analgesia, their β-arrestin-mediated side effects contrast with CB_2_’s G protein-biased anti-inflammatory actions. Similarly, adenosine receptors (A_2A_R and A_3_R) modulate chondrocyte homeostasis through divergent pathways — cAMP vs. β-arrestin — highlighting the need for receptor-specific bias profiling. However, critical gaps persist, such as the underexplored role of chemokine receptor dimerization in OA progression and species-specific ligand biases that hinder translational relevance.

Emerging trends underscore the synergy between natural products and precision pharmacology. Terpenoids (e.g., paeoniflorin) and flavonoids (e.g., biochanin A) exhibit multi-target efficacy but require structural optimization to overcome poor bioavailability. Advances in AI-driven molecular design and cryo-EM are accelerating the discovery of allosteric modulators with refined bias profiles. Future research must prioritize mechanistic depth (e.g., resolving GPCR-ligand complexes), translational rigor (e.g., OA models mimicking human heterogeneity), and clinical integration through biomarker-driven patient stratification. Interdisciplinary efforts combining TCM insights with synthetic biology and machine learning will be pivotal in unlocking next-generation therapies that balance efficacy with long-term joint homeostasis.
